# Potential impact of NICE guidelines on referrals from primary care to nephrology: a primary care database and prospective research study

**DOI:** 10.3399/BJGP.2022.0145

**Published:** 2022-11-15

**Authors:** Michael K Sullivan, Bhautesh Dinesh Jani, Elaine Rutherford, Paul Welsh, Alex McConnachie, Rupert W Major, David McAllister, Dorothea Nitsch, Frances S Mair, Patrick B Mark, Jennifer S Lees

**Affiliations:** Institute of Cardiovascular and Medical Sciences, University of Glasgow, Glasgow.; General Practice and Primary Care, Institute of Health and Wellbeing, University of Glasgow, Glasgow.; Institute of Cardiovascular and Medical Sciences, University of Glasgow, Glasgow; consultant in renal medicine, Renal Unit, Mountainhall Treatment Centre, NHS Dumfries and Galloway, Dumfries.; Institute of Cardiovascular and Medical Sciences, University of Glasgow, Glasgow.; Robertson Centre for Biostatistics, Institute of Health and Wellbeing, University of Glasgow, Glasgow.; Department of Cardiovascular Sciences, University of Leicester, Leicester; consultant nephrologist, John Walls Renal Unit, University Hospitals of Leicester, Leicester.; Public Health, Institute of Health and Wellbeing, University of Glasgow, Glasgow.; Faculty of Epidemiology and Population Health, London School of Hygiene & Tropical Medicine, London.; General Practice and Primary Care, Institute of Health and Wellbeing, University of Glasgow, Glasgow.; Institute of Cardiovascular and Medical Sciences, University of Glasgow, Glasgow.; Institute of Cardiovascular and Medical Sciences, University of Glasgow, Glasgow.

**Keywords:** diagnosis, general practice, guidelines, hospital referrals, large database research, renal medicine

## Abstract

**Background:**

National Institute for Health and Care Excellence 2021 guidelines on chronic kidney disease (CKD) recommend the use of the Kidney Failure Risk Equation (KFRE), which includes measurement of albuminuria. The equation to calculate estimated glomerular filtration rate (eGFR) has also been updated.

**Aim:**

To investigate the impact of the use of KFRE and the updated eGFR equation on CKD diagnosis (eGFR <60 mL/min/1.73 m^2^) in primary care and potential referrals to nephrology.

**Design and setting:**

Primary care database (Secure Anonymised Information Linkage Databank [SAIL]) and prospective cohort study (UK Biobank) using data available between 2013 and 2020.

**Method:**

CKD diagnosis rates were assessed when using the updated eGFR equation. Among people with eGFR 30–59 mL/min/1.73 m^2^ the following groups were identified: those with annual albuminuria testing and those who met nephrology referral criteria because of: a) accelerated eGFR decline or significant albuminuria; b) eGFR decline <30 mL/ min/1.73 m^2^ only; and c) KFRE >5% only. Analyses were stratified by ethnicity in UK Biobank.

**Results:**

Using the updated eGFR equation resulted in a 1.2-fold fall in new CKD diagnoses in the predominantly White population in SAIL, whereas CKD prevalence rose by 1.9-fold among Black participants in UK Biobank. Rates of albuminuria testing have been consistently below 30% since 2015. In 2019, using KFRE >5% identified 182/61 721 (0.3%) patients at high risk of CKD progression before their eGFR declined and 361/61 721 (0.6%) low-risk patients who were no longer eligible for referral. Ethnic groups ‘Asian’ and ‘other’ had disproportionately raised KFREs.

**Conclusion:**

Application of KFRE criteria in primary care will lead to referral of more patients at elevated risk of kidney failure (particularly among minority ethnic groups) and fewer low-risk patients. Albuminuria testing needs to be expanded to enable wider KFRE implementation.

## INTRODUCTION

In 2006, guidelines on chronic kidney disease (CKD) encouraged the use of estimated glomerular filtration rate (eGFR) to identify and categorise CKD.^1^^,^^2^ Laboratories started routinely reporting eGFR and criteria for referral to nephrology were outlined: eGFR <30 mL/min/1.73 m^2^, accelerated decline in eGFR, and significant albuminuria.

Only 1%–4% of people with CKD will progress to kidney failure requiring treatment (that is, dialysis or kidney transplantation).^3^^,^^4^ If GPs can identify high- risk people and refer them before they progress to this late stage, nephrologists may be able to slow progression to kidney failure and/or prepare for dialysis or transplantation.

In 2021, National Institute for Health and Care Excellence (NICE) guidelines made two recommendations that influence CKD diagnosis and referral to nephrology.^5^ Although referral criteria based on accelerated eGFR decline and albuminuria were unchanged, using an eGFR threshold of 30 mL/min/1.73 m^2^ was replaced by the Kidney Failure Risk Equation (KFRE).^6^ This allows an individual’s risk of kidney failure to be defined, permitting referral and management in secondary care to be personalised to their future risk, rather than based on any particularly eGFR value. The KFRE equation takes account of four variables:

- age;
- sex;
- eGFR; and
- urine albumin-to-creatinine ratio (uACR).

GPs are advised to refer to nephrology if the 5-year risk of kidney failure is >5%.

The NICE guidelines also recommend that laboratories use eGFR equations that do not include ethnicity. The inclusion of ethnicity in these equations has been debated for several years, with many concerned that ethnicity is a social construct rather than a biological one.^7^

**Table:** How this fits in

The National Institute for Health and Care Excellence 2021 guidelines on chronic kidney disease (CKD) recommended GPs use the Kidney Failure Risk Equation (KFRE) to guide referrals to nephrology. The equation used for estimating glomerular filtration rate (eGFR) has been updated. In a Welsh primary care population (Secure Anonymised Information Linkage Databank), there was a 1.2-fold fall in new CKD diagnoses if laboratories used the updated equation to report eGFR. New CKD referral criteria identify patients at high risk of CKD progression before their eGFR falls below 30 mL/min/1.73 m^2^, particularly among minority ethnic groups. Implementation of KFRE will rely on raising awareness among GPs and improved albuminuria testing rates, which are currently low.

The updated equation for eGFR calculation (which does not include ethnicity) was released in 2021,^8^ shortly after the updated NICE guideline recommended that such equations be used. A nationally representative primary care cohort followed by a prospective research cohort were studied to assess the potential impact of these guideline changes on diagnosis of CKD in primary care, potential referral rates to nephrology, and whether there was a differential impact in ethnic groups.

## METHOD

### Data sources

The Secure Anonymised Information Linkage Databank (SAIL) is an electronic health records repository that holds primary care data for 79% of the population of Wales.^9^

Patients aged >18 years with serum creatinine values available between 1 January 2013 and 31 December 2020 are included in this study. Values before 2013 were not included because non-validated laboratory analysers may have been in use.^10^ Creatinine, uACR values, and diagnoses of CKD, diabetes mellitus, and hypertension were taken from GP Read codes, as previously described.^11^ Ethnicity is not consistently recorded in these records, so it was not used.

UK Biobank is a research cohort that enrolled volunteer participants between 2006 and 2010 as previously described.^12^^,^^13^ Sociodemographic and medical information were self-reported and blood and urine samples were taken at baseline.^13^ The sampling process has been described previously.^14^^–^^16^

In brief, serum and spot urine samples were collected and analysed at an accredited central laboratory. Creatinine was measured using a Beckman Coulter AU5800 analyser and the assay was externally quality controlled. Of 502 536 participants initially recruited, in this study 76 were excluded as they withdrew ongoing consent for follow- up, as well as 33 144 participants with missing creatinine values at baseline, 2205 with unknown ethnicity, and 550 with kidney failure requiring replacement therapy at baseline.

### Albuminuria testing

From SAIL data, for this study, patients were identified with prevalent CKD stage G3A/G3B, that is, two eGFR values^8^ 30–59 mL/ min/1.73 m^2^ ≥3 months apart with no values >60 mL/min/1.73 m^2^^.^^17^

Prevalence of CKD stage G3A/G3B was described by calendar year from 2013 to 2020. Rates of annual uACR testing were established for these patients. This included patients with previously identified CKD, so long as they were alive throughout the year. uACR testing was stratified by age, sex, eGFR, diabetes mellitus, and hypertension, and whether CKD was coded in their medical records. Tested and untested groups were compared using χ^2^-tests for categorical variables and analysis of variance tests for continuous variables.

From UK Biobank data, participants were labelled with CKD stage G3A/G3B if the single baseline eGFR available was 30–59 mL/min/1.73 m^2^.

### Patients meeting referral criteria to nephrology

From the group of patients with CKD stage G3A/G3B, those eligible for nephrology referral were identified using three criteria.

- *Accelerated eGFR decline or albuminuria*: eGFR decline by ≥25% or ≥15 mL/min/1.73 m^2^ within 12 months, sustained for ≥3 months with no values rising above the threshold; or uACR ≥70 mg/mmol. Two sensitivity analyses were performed: a) patients with known diabetic nephropathy are not to be referred with this albuminuria threshold. The authors of the current study therefore studied whether patients with diabetes would only be referred if they had heavy to nephrotic-range proteinuria (uACR >200 mg/mmol); and b) haematuria criteria were not included because information on haematuria was not available. The authors of the current study therefore studied the impact on referrals when using a lower uACR threshold of 30 mg/mmol instead of 70 mg/mmol, which would warrant nephrology referral if accompanied by haematuria.
- *eGFR* < *30 only *: incident eGFR <30 mL/ min/1.73 m^2^ sustained for ≥3 months with no values rising above 30.
- *KFRE* > *5% only *: 5-year KFRE of >5%.^5^^,^^6^^,^^19^ For patients with several measurements of eGFR and uACR, the highest KFRE result was selected, given GPs are likely to act on an elevated result when it becomes available.

Those currently or previously under nephrology follow-up were discounted, assuming that patients discharged back to primary care had already been assessed. The patients eligible for referral based on eGFR <30 only and KFRE >5% only were compared using analysis of variance tests for age and χ^2^-tests for sex (excluding those eligible by both criteria).

From UK Biobank data, participants with 5-year KFRE >5% were identified and the results stratified, first by ethnicity and then by ethnicity and diabetes mellitus status.

### CKD prevalence according to eGFR equation

CKD prevalence in SAIL was estimated using different eGFR equations and stratified by ethnicity in UK Biobank. The approach is detailed in the Supplementary Appendix S1. Statistical analyses were conducted using R (version 4.1.3).

## RESULTS

In total, 1 845 040 patients in SAIL and 466 561 participants in UK Biobank were studied (Table 1). Supplementary Figure S1 provides details of the people included in the analysis in a consort diagram.

**Table 1.** Summary of baseline characteristics by cohort

**Characteristic**	**SAIL**	**UK Biobank**
***n***	1 845 040	466 561

**Female sex, *n* (%)**	1 015 078 (55.0)	253 258 (54.3)

**Age, years, median (IQI)**	53 (37–67)	58 (50–63)

**Ethnicity, *n* (%)^a^**		
White		441 848 (94.7)
Black		8241 (1.8)
Asian		11 293 (2.4)
Mixed		1013 (0.2)
Other		4166 (0.9)

**Diabetes mellitus, *n* (%)**	310 352 (16.8)	24 241 (5.2)

**Hypertension, *n* (%)**	569 918 (30.9)	111 475 (23.9)

a *Ethnicity was not consistently recorded in SAIL. The Welsh population is approximately 95.0% White.^18^ IQI = interquartile interval. SAIL = Secure Anonymised Information Linkage Databank.*

### Albuminuria testing

**Figure 1.**
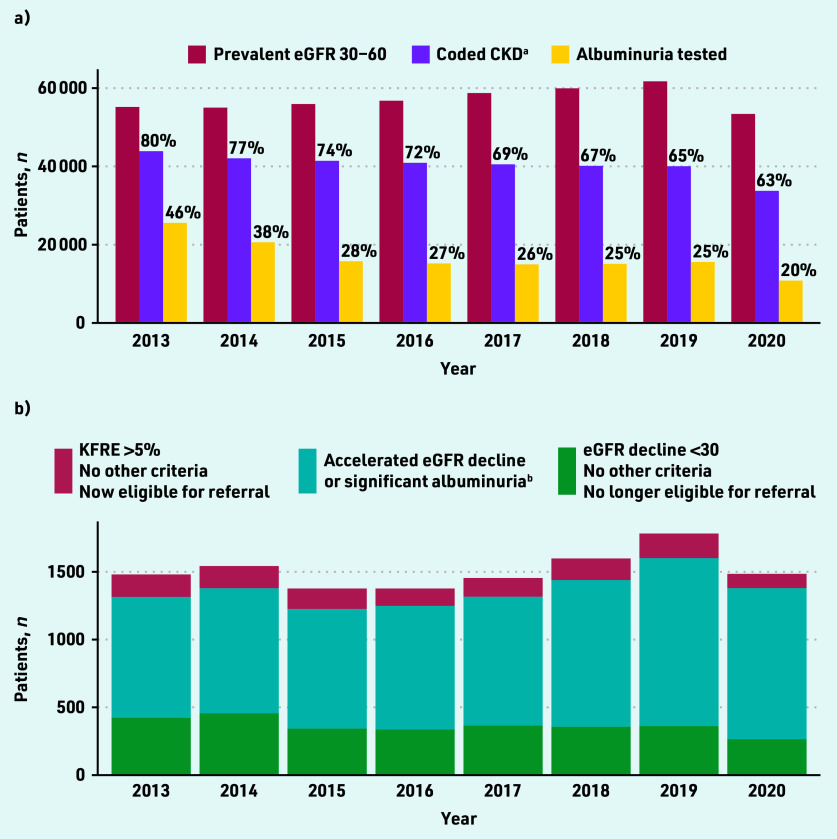
*Prevalent CKD stage G3A/G3B in SAIL 2013–2020. a) Monitoring; and b) potential referrals to renal clinic. ^a^In the UK, GPs are expected to add a CKD code as an entry to the patient diagnosis list. The denominator for percentages in ‘a)’ are those with prevalent eGFR 30–60 mL/min/1.73 m^2^. ^b^ Some of these patients also have KFRE >5% or eGFR decline <30 mL/ min/1.73 m^2^, but their identification is unchanged by the guideline change. CKD = chronic kidney disease. eGFR = estimated glomerular filtration rate. KFRE = Kidney Failure Risk Equation. SAIL = Secure Anonymised Information Linkage Databank.*

In SAIL, the prevalence of CKD stage G3A/G3B among those with a creatinine measurement was 55 193/752 517 (7.3%) in 2013 and 61 721/850 394 (7.3%) in 2019 (Figure 1a). In 2020, spanning the COVID-19 pandemic, this figure was 53 406/659 740 (8.1%). In UK Biobank, there were 6739/466 561 (1.4%) participants with CKD stage G3A/G3B (data not shown).

Among patients with prevalent CKD stage G3A/G3B in SAIL, the rate of annual uACR testing fell from 46.3% in 2013 to 25.3% in 2019 (Figure 1a). eGFR and uACR testing were reduced further in 2020 during the COVID-19 pandemic.

The demographics of patients with coded and biochemical CKD (that is, a CKD code in their records, and eGFR 30–59 mL/min/1.73 m^2^) G3A/G3B (56.1% female, median age 82 years, interquartile interval (IQI) 76–88) were similar to patients with biochemical CKD but not coded (that is, no CKD code in their records, but eGFR 30–59 mL/min/1.73 m^2^; 55.1% female, median age 81 years, IQI 75–87). Of the patients with CKD G3A/G3B at any time, factors associated with higher rates of uACR testing were male sex, young age (the tested group were a median of 2 years younger), coded CKD, diabetes mellitus, hypertension, and low eGFR (see Supplementary Table S1 and Supplementary Figures S2 and S3).

### Patients meeting referral criteria to nephrology

#### Patients meeting referral criteria based on accelerated eGFR decline or albuminuria

In SAIL, between 883 and 1240 people per year had accelerated eGFR decline or significant albuminuria (Figure 1b). Referral of these people was unaffected by the guideline change. In the sensitivity analysis using a uACR threshold of 200 mg/mmol for patients with diabetes led to a reduction of patients eligible for referral by these criteria (see Supplementary Figure S4). Using a uACR threshold of 30 mg/mmol instead of 70 mg/ mmol led to an increase in patients eligible for referral by these criteria, assuming these patients also had evidence of haematuria (see Supplementary Figure S5).

#### Patients meeting referral criteria based on eGFR < 30 only

In SAIL, between 265 and 456 people per year developed CKD G4–5 without accelerated eGFR decline or significant albuminuria (0.5% to 0.8% of those with CKD stage G3A/G3B; Figure 1b). In 2019 (the last year before the COVID- 19 pandemic), this figure was 361/61 721 (0.6%). Using the 2021 NICE guidelines, these patients would no longer necessarily be eligible for referral.

#### Patients meeting referral criteria based on KFRE > 5% only

In SAIL, KFRE consistently identified a substantial number of patients who should be referred to nephrology who did not meet other criteria (Figure 1b). In 2019 (the last year before the COVID-19 pandemic), 182/61 721 (0.3%) patients with CKD stage G3A/G3B met referral criteria by KFRE only. In the same year, 802/17 332 (4.6%) patients with CKD stage G3A/G3B and uACR measured had KFRE >5% (this figure includes those already under nephrology follow-up). Importantly, patients eligible for referral only by KFRE criteria were a median of 5 years younger and more likely to be males than those eligible by eGFR <30 only (see Supplementary Table S2, *P*<0.001 for both variables).

**Figure 2.**
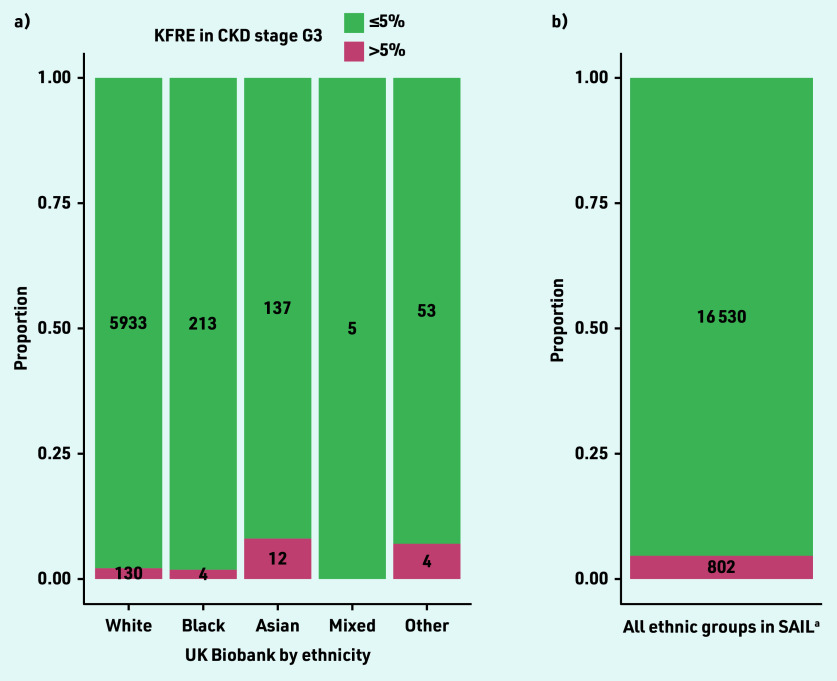
*Kidney failure risk by a) ethnicity in UK Biobank; and b) overall in SAIL. ^a^Ethnicity is not consistently recorded in SAIL. CKD = chronic kidney disease. eGFR = estimated glomerular filtration rate. KFRE = Kidney Failure Risk Equation. SAIL = Secure Anonymised Information Linkage Databank.*

In UK Biobank, 150/6739 (2.2%) participants with eGFR 30–59 mL/ min/1.73 m^2^ had a 5-year KFRE >5%. The proportion of participants with KFRE >5% was highest in those of Asian and other ethnicity (Figure 2). In both cohorts and in all ethnic groups, the proportion of participants with elevated KFRE was higher among those with diabetes mellitus compared with those without diabetes mellitus (see Supplementary Figure S6).

### CKD prevalence according to eGFR equation

eGFR was lowest using the Modification of Diet in Renal Disease equation (eGFR_MDRD_) and highest using the 2021 version of the Chronic Kidney Disease Epidemiology Collaboration (eGFR_2021_) equation (see Supplementary Figure S7). Among patients in SAIL with CKD G3–5, a switch to using eGFR_2021_ from eGFR_MDRD_ would be associated with an average increase in reported eGFR of 2.7 mL/min/1.73 m^2^ (IQI 1.2–4.5).

**Figure 3.**
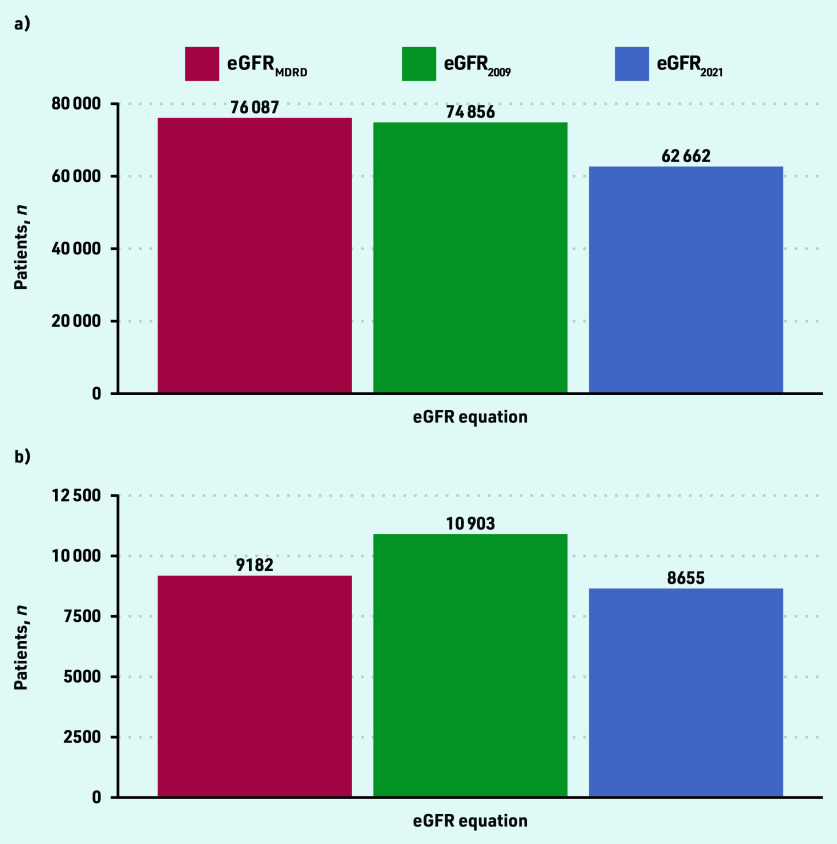
*Numbers of patients with incident CKD in SAIL using different eGFR equations between 2013 and 2020. a) eGFR <60 mL/min/1.73 m^2^; and b) eGFR <30 mL/min/1.73 m^2^. CKD = chronic kidney disease. eGFR = estimated glomerular filtration rate. SAIL = Secure Anonymised Information Linkage Databank.*

In practice, this change to laboratory reporting from eGFR_MDRD_ to eGFR_2021_ would result in a 1.2-fold reduction in people diagnosed with CKD stage G3–5 and a 1.1- fold reduction in CKD G4–5. The number of patients in SAIL with incident CKD stage G3–5 or incident CKD stage G4–5 was lowest using the updated eGFR_2021_ equation (Figure 3). In general, a change in laboratory reporting of eGFR_MDRD_ to eGFR_2021_ is more likely to de-classify older females from a CKD diagnosis (see Supplementary Table S3).

In UK Biobank, the changes in eGFR values and thus categorisation of CKD using different equations were similar among non-Black participants (see Supplementary Figure S8 and Supplementary Table S4). If laboratories reported eGFR_2021_ instead of eGFR_MDRD_, the number of non-Black participants meeting criteria for CKD G3–5 diagnosis would increase from 124 to 237 (a 1.9-fold increase). This would be offset by 3463/10 225 (33.9%) fewer non-Black participants classified as having CKD G3–5 if making this same reporting change. If eGFR_2021_ was used to calculate KFRE instead of eGFR_MDRD_, the number of Black participants with a KFRE >5% would increase from three to four individuals.

## DISCUSSION

### Summary

Implementation of KFRE in primary care can help GPs identify a small proportion of additional patients with previously unrecognised elevated risk who could then be referred to nephrology before their eGFR declines below 30 mL/min/1.73 m^2^. This will be offset by reductions in referrals for patients with slow declines in their eGFR. Regular uACR testing is required for all patients with CKD for KFRE calculation, but annual uACR testing is not consistently performed in all people with CKD. Optimal referrals to nephrology will therefore depend on increases in uACR testing and awareness among GPs of KFRE.

### Strengths and limitations

This study has some limitations. First, the rates of observed kidney failure events are not reported. Neither SAIL nor UK Biobank are linked to national renal registry records, which most accurately record kidney failure events. However, KFRE has already been validated extensively,^6^^,^^20^ and is now recommended as a tool to guide risk assessment and nephrology referral, and to aid with risk reduction strategies. Second, the lack of ethnicity data in SAIL means this key information could not be reported. Although ethnicity differences in KFRE could be reported for the UK Biobank, diagnosis of CKD here was based on a single baseline measure of kidney function, and it was not possible to use two confirmatory measurements at least 3 months apart, as recommended in clinical guidelines.^17^ Given the relatively healthy and predominantly White population in UK Biobank,^21^ and lower rate of CKD and heavy albuminuria observed than in the general population, it is likely that use of the KFRE underestimated risk of kidney failure in the general population.

Finally, a uACR of >200 mg/mmol was used as an equivalent to nephrotic-range proteinuria (that is, urine protein to creatinine ratio [uPCR] of >300 mg/mmol). Although uACR 200 mg/mmol is approximately equivalent to uPCR 300 mg/mmol,^22^ it would have been preferable if uPCR data had been available to identify patients with nephrotic- range proteinuria.^23^

### Comparison with existing literature

Reduced rates of laboratory testing in primary care in the UK have been reported before in association with changes in the Quality and Outcomes Framework.^24^ The incentivisation of annual uACR testing for people with diabetes mellitus was removed from the GP contract in April 2014.^25^

The 2017 National CKD Audit in England and Wales reported that 31.1% of people with CKD had an annual uACR test.^26^ In 2017 in the current study the rate was 25.5%, which may have been lower because the CKD audit reports on stages G3–5, whereas this study was limited to stage G3A/G3B. In keeping with the findings of the National CKD Audit, the current study suggests that improvements in albuminuria testing particularly need to be targeted in patients with non-diabetic CKD.

By improving uACR testing and using KFRE, GPs will identify patients at high risk of kidney failure at a younger age and an earlier stage of CKD. The lifetime risk of kidney failure is higher at a younger age and patients are more likely to live long enough to require dialysis or kidney transplantation compared with older adults (such as those over the age of 80 years). However, CKD is associated with broader risks, primarily cardiovascular disease. Greater recognition of patients at risk of progressive CKD will also identify people at higher risk of cardiovascular disease^27^^,^^28^ and provide earlier opportunities for primary prevention interventions such as statins and sodium- glucose co-transporter-2 inhibitors.^29^^,^^30^

A study using 5-year KFRE >3% to guide nephrology referrals similarly found that using KFRE to guide referrals instead of an eGFR threshold of 30 mL/min/1.73 m^2^ would would lead to more patients at elevated risk of kidney failure being followed up in secondary care and more low-risk patients being monitored in primary care.^31^

Importantly, KFRE was more likely to be elevated in ethnic groups other than White, in keeping with the previous literature suggesting that Black and minority ethnic groups are at the greatest risk of CKD progression.^32^ The current study did not find high rates of elevated KFRE in Black participants, which may reflect low levels of albuminuria, predominance of White participants, and healthy volunteer bias in UK Biobank.^21^

Nevertheless, the current study shows that implementation of KFRE can identify those at the highest risk and may go some way to attenuating healthcare inequalities in CKD management in Black and minority ethnic groups. Although the UK population is predominantly White, the current study confirms that among people of Black and minority ethnicity, the rates of CKD will rise significantly if laboratories start to report eGFR_2021_ using the 2021 CKD-EPI 2021 equation.

### Implications for research and practice

At stage G3A/G3B CKD, only a small proportion of patients are at high risk of CKD progression. These patients can be readily identified using KFRE; however, KFRE will only report elevated 5-year risk of kidney failure if there is at least moderate detected albuminuria (that is, uACR >3 mg/mmol).

Low uACR testing rates (consistently <30%) were observed in primary care from 2015 onwards, and particularly low rates in 2020, probably because much routine disease monitoring stopped during the COVID-19 pandemic. Annual albuminuria testing rates are reported in the current study, although Kidney Disease: Improving Global Outcomes (KDIGO) guidelines recommend more frequent testing: 4- or 6-monthly in those with eGFR 30–44 mL/ min/1.73 m^2^ and in those with uACR >3 mg/mmol.^17^ The current study may therefore have overestimated the proportion of individuals with albuminuria testing appropriate for their stage of CKD.

Several factors may explain the suboptimal rates of albuminuria testing seen in these populations with CKD in this study. Many people with CKD are unaware of the diagnosis (10%–59%^33^^,^^34^), perhaps because most of them are asymptomatic. By raising patient awareness of CKD and the importance of albuminuria testing patients may engage better with CKD management, for example, by encouraging home blood pressure monitoring and proactively providing urine samples for albuminuria testing.

Higher rates of albuminuria testing in this study were found in people with coded compared with biochemical CKD. As clinical and laboratory records are predominantly electronic, there may be a role for automating KFRE calculation or indeed entry onto CKD registers, which has been associated with improved albuminuria testing.^35^

In summary, use of KFRE in general practice will identify more patients with CKD at risk of kidney failure and needing nephrology referral, particularly among minority ethnic groups. Fewer patients will be referred with eGFR declining slowly to <30 mL/min/1.73 m^2^ with no markers of elevated risk. However, KFRE use will be dependent on universal and regular uACR testing, particularly among older adults and those without diabetes mellitus. Overall, updated eGFR equations, if used by UK laboratories, will reduce the numbers of patients categorised as having CKD.

## Data

Data that support the findings of this study are available from UK Biobank and SAIL, subject to successful registration and application process. Further details can be found at https://www.ukbiobank.ac.uk and https://saildatabank.com
